# Vibrational Stress Analysis and Test Verification of Satellite Honeycomb Sandwich Plate Subjected to Acoustic Excitation

**DOI:** 10.3390/s25247444

**Published:** 2025-12-07

**Authors:** Liang Zhang, Yousheng Shi, Shanbo Chen, Xiangyu Zhao, Jisong Yu, Lei Zhang

**Affiliations:** 1 Chang Guang Satellite Technology Co., Ltd., Changchun 130000, China; zhangliang@jl1.cn (L.Z.); shiyousheng@jl1.cn (Y.S.); ak48css@sina.com (S.C.); zhaoxiangyu@jl1.cn (X.Z.); yujisong@jl1.cn (J.Y.); 2 Department of Precision Instrument, Tsinghua University, Beijing 100084, China; 3 Changchun Institute of Optics, Fine Mechanics and Physics, Chinese Academy of Sciences, Changchun 130033, China; 4 University of Chinese Academy of Sciences, Beijing 100049, China

**Keywords:** acoustic excitation, random vibration, response and stress, honeycomb sandwich plate

## Abstract

The vibrational response and stress of satellites subjected to acoustic excitation are essential components to consider in the design process of large satellite constructions. To precisely forecast the vibrational response and stress subjected to acoustic excitation of a large satellite honeycomb sandwich plate, this article employs finite element modeling software to create a finite element model of satellite, equivalent honeycomb panels to orthotropic shear plates of identical stiffness and dimensions, and convert the acoustic excitation into random pressure on the surface of the flat plate, applying it to the surface of the satellite, then the vibration response and stress analysis subjected to acoustic excitation can be performed. The honeycomb structure collapsed after noise testing on a specific model of a large satellite, the aforementioned technique for response and stress validation was employed: the stress simulation analysis revealed that the maximum shear stress at the fracture site of the honeycomb panel was 0.45 MPa, exceeding the ultimate stress value of 0.44 MPa for the sparse honeycomb core at that location, leading to the collapse and fracture of the honeycomb panel. To resolve this matter, altering the honeycomb core design framework, a local dense honeycomb structure was implemented. The simulated shear stress of the honeycomb core is 0.54 MPa, which is below the stress limit of 2.43 MPa for the dense honeycomb core. To verify the feasibility of the plan, the noise test was conducted once again. Owing to the incapacity to test the shear stress of the honeycomb core, conducted strain testing on the surface of the honeycomb collapse and derive stress results by calculation, the test results deviate from the modeling values by 8.92%. And the maximum discrepancy between the simulated noise response and the experimental noise response is 0.58 g, validated the efficacy and precision of the simulation method, and successfully resolved the issue of damage to satellite honeycomb panels. This simulation method can precisely forecast the vibrational response and stress of satellites subjected to acoustic excitation.

## 1. Introduction

Along with the rapid development of aerospace technologies, to satisfy the need for in-orbit utilization, increasing load size, satellite size, mass, and the structure of satellite tends to be complicated. During the execution of evaluations and quality audits on larger satellites, noise test is required. The mechanical conditions of satellites during launch are relatively stringent, primarily for two reasons. One factor is the force conveyed through the docking interface of the satellite and rocket, including the relatively stable mechanical conditions produced by engine thrust and the transient mechanical conditions resulting from rocket engine ignition, stage separation, etc. The second factor pertains to the mechanical conditions directly affecting the satellite’s surface caused by the noise environment within the fairing, which must be considered in the satellite design process due to its impact on the satellite structure. Considering all of the above factors, it is essential to analyze the response and stress of large satellites subject to acoustic excitation. In previous research, there have been many studies on the response analysis of thin-walled structures subjected to noise excitation. The stress analysis and structural strength verification of satellites subjected to acoustic excitation remain essential in the design of large satellite structures and require particular attention.

Previous investigators have performed comprehensive studies on the response analysis of noise excitation. The prevalent methodologies mostly include finite element analysis (FEA), statistical energy analysis (SEA) [1], and the FE-SEA hybrid approach. Finite Element Analysis (FEA) models serves as a fundamental methodology in aerospace engineering for structural design and performance optimization. By discretizing complex spacecraft structures into a finite set of elements, this analytical technique employs numerical simulations to predict structural mechanical responses under diverse operational conditions, thereby ensuring design reliability and safety. A systematic model verification strategy consisted of finite element modeling, modal test, correlation analysis, and model updating was proposed [2]. Li Xiufeng [3] proposed a method of finite clement model simplification based on topology optimization aiming at the difficult modeling problem of dynamic analysis model caused by complex composition of spacecraft equipment. Finite element analysis offers the benefit of calculating the response at any location inside the structure and obtaining the distribution of that response. Nevertheless, as the frequency of analysis escalates, the increase in grid density and the rise in uncertainty aspects inside the structure will significantly elevate computational costs and reduce analytical accuracy. Consequently, it is inadequate for predicting high-frequency vibration responses. Du Ligang [4] performed modal analysis of a specific aircraft in a free-free state and conducted random vibration response study under aerodynamic noise utilizing Nastran, thereby validating the efficacy of the finite element model for predicting vibration environments. Li Qing and Xing Likun [5] employed modal analysis to examine the random vibration response of cabin panels subjected to noise loads, including issues such as damping parameters and the effects of additional mass in the flow field. Cao Maoguo and Li Lin [6] presented a calculation formula and methodology for the power spectral density of the random vibration acceleration response of thin-walled cylindrical shell structures subjected to noise loads, and derived the calculation formula for the power spectral density and mean square value of the Von Mises stress response, which can be directly applied in fatigue strength analysis; random vibration analysis is primarily employed to evaluate the dynamic response of a spacecraft that is subjected to random base excitation or acoustic excitation. Chen Hua [7] discussed the fatigue damage analysis of a spacecraft structure under random vibration; Jie Zhang [8] performed an equivalency study on spacecraft acoustic excitation and random vibration; Dulat Akzhigitov [9] presents the results of numerical modeling and vibration testing of a nanosatellite’s optical payload, aimed at assessing its mechanical stability under the mechanical impacts of launch. Javier Sanz-Corretge [10] presents a spectral-based methodology for the probabilistic failure analysis of composite laminates subjected to zero-mean, stationary Gaussian random vibrations. Statistical energy analysis (SEA) promises theoretical advantages in predicting high-frequency vibrational responses. Considering the unpredictability of structural connection methods and manufacturing processes, complex and specific models are not required for the effective computation of statistical average vibration response values [11,12,13,14]. As a result, a reply and response distribution at defined regions cannot be provided, leading to insufficient computational accuracy in the low to mid frequency range. Wang Dong and Liu Wei [15] examined the attributes of several dynamic environment prediction techniques, emphasizing the concepts and applications of statistical energy analysis (SEA) methodologies. They confirmed that precise assessment of SEA parameters in a single experiment can more accurately predict dynamic results. Zhang Jin, Zou Yuanjie [16] employed the FE-SEA hybrid approach to analyze more intricate coupled systems utilizing conventional finite element methods and statistical energy analysis. Zhu Weihong and Han Zengyao [17] employed the Finite Element Energy Statistical Analysis (FE-SEA) method to develop a spacecraft acoustic vibration prediction model, subsequently comparing it with experimental data to validate the efficacy of the prediction approach. Peng Wang [18] analyzed vibration and reliability analysis of non-uniform composite beam under random load; Jesús M. [19] allowed us to determine the fatigue life of a component that is being subjected to a random vibration environment. Koki SATO [20] proposed a new modal analysis method and evaluated its effectiveness compared to acoustic results from experiments. Dong, F [21] analyzed the fatigue life of honeycomb sandwich panels and conducted experiments.

Currently, most of the large satellite structures employ composite materials, particularly honeycomb panels. Specialized methods are necessary for stress analysis of the satellite’s structure in this configuration, owing to the absence of relevant elements in finite element software. The primary application technique is the equivalent method [22]; Xu Shengjin and Kong Xianren [23] indicated an equivalent approach for orthotropic honeycomb sandwich panels based on low-order shear theory, validating its accuracy by experimental verification. He Rui [24] posited that aluminum honeycomb sandwich panels demonstrate a nonlinear frequency response function in random vibration tests, characterized by a reduction in FRF amplitude and an elevation in damping ratio as the excitation intensity increases, aligning with the nonlinear damping model; Laurent Wahl [25] analyzed shear stresses in honeycomb sandwich plates, some equations are derived in order to calculate the real shear stresses from the shear stresses of the homogeneous core. Zhao Jingjing [26] presents the intrinsic nature of an adhesively bonded multi-layer structure which increases the risk of debonding when the honeycomb sandwich structure is under strain or exposed to varying temperatures. Wang Mo-Nan [27] focuses on the in-plane shear respond and failure mode of large size honeycomb sandwich composites which consist of plain weave carbon fabric laminate skins and aramid paper core. Alaa Al-Fatlawi [28] investigated replacing the currently used aluminum base plates of aircraft pallets with composite sandwich plates to reduce the weight of the pallets, thereby the weight of the unit loads transported by aircraft. Qi, D.Z [29] analyzed the buckling loads of a composite sandwich structure, which was reinforced by a honeycomb layer and filled with viscoelastic damping material, Fei-Hao Li [30] established a three-dimensional vibration theory for ultralight cellular-cored sandwich plates subjected to linearly varying in-plane distributed loads; Zhuo Xu [31] introduced a nonlinear damping prediction model for partially filled composite honeycomb sandwich panels and formulated a structural energy equation by integrating high-order shear theory with finite element analysis, thereby offering a more precise computational approach for examining the nonlinear damping properties of honeycomb sandwich structures. Sadiq Emad Sadiq [32] presents a suggested analytical solution for a forced vibration of an aircraft sandwich plate with a honeycomb core under transient load.

Based on a previous study, this article develops a finite element model of a large satellite with honeycomb sandwich panels that are equivalent to orthotropic shear plates of identical stiffness and sizes, and calculated the elastic modulus, shear modulus, Poisson’s ratio, and equivalent density of the mechanical model, providing parameters for stress calculation. This article abandons the traditional method of noise excitation analysis, transforms the sound pressure spectrum into pressure power spectral density, converts noise excitation into random excitation, and implements it on the surface of the satellite finite element model. In previous analyses, the damping ratio for frequency response analysis was generally taken as a constant value of 0.03, and in order to obtain a more accurate analysis, a variable damping model was adopted, analyzing the vibration response and stress subjected to acoustic excitation, conducting satellite noise testing, and then comparing it with noise test results to validate the efficacy of the predictive method.

## 2. Principles of Simulation Analysis

### 2.1. Equivalent Honeycomb Sandwich Panel

In the finite element analysis of honeycomb panels, an identical method to the honeycomb structure is essential, with the primary theories employed including sandwich panel theory and honeycomb panel theory.

The sandwich panel theory [33] assumes that the honeycomb layer can resist lateral shear deformation and possesses a certain in-plane stiffness. The upper and lower skins conform to the Kirchhoff hypothesis, ignoring their capacity to resist lateral shear stress. The honeycomb core is analogous to an uniform orthotropic layer of constant thickness.

The equivalent elastic characteristics of the hexagonal honeycomb employed in this paper are as follows.

(1) $$E_{x}=E_{y}=\frac{4}{\sqrt{3}}{\left(\frac{t}{l}\right)}^{3}E$$

(2) $$G_{xy}=\frac{\sqrt{3}\gamma }{2}{\left(\frac{t}{l}\right)}^{3}E,G_{xz}=\frac{\gamma }{\sqrt{3}}\frac{t}{l}G$$

(3) $$G_{yz}=\frac{\sqrt{3}\gamma }{2}\frac{t}{l}G,\nu_{xy}=\frac{1}{3}$$

where E, G represents mechanical characteristics of sandwich composites; ν represents Poisson’s ratio; l denotes the length, and t signifies the thickness of the honeycomb cell wall panel; γ is the correction factor, dependent upon the technique, typically ranging from 0.4 to 0.6, in this work there was a correlation factor of γ = 0.5, with a theoretical value of 1.0.

**Figure 1:**
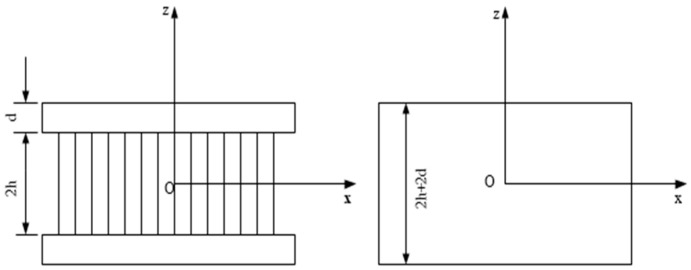
Equivalent schematic diagram.

The honeycomb panel theory posits that the full honeycomb sandwich panel can be represented as an orthotropic panel of equivalent stiffness and dimensions. Figure 1 illustrates an orthotropic honeycomb sandwich panel with uniform geometric dimensions alongside its comparable panel. The surface thickness of the honeycomb panel is d, the core height is 2h, and d is significantly less than 2h. The total thickness of the equivalent panel is 2h+2d.

The cross-sectional displacement of honeycomb panels exhibits continuity, and in accordance with the low-order shear theory, this displacement must conform to the following conditions:

(4) $$\left\{\begin{array}{l} u\left(x,y,z,t\right)=z\varphi_{x}\left(x,y,t\right)\\ v\left(x,y,z,t\right)=z\varphi_{y}\left(x,y,t\right)\\ w\left(x,y,t\right)=w\left(x,y,t\right) \end{array}\right.$$

where u, v and w represent displacement components; $\varphi_{x}$ and $\varphi_{y}$ denote the degrees of rotation of the normal outside the central plane around the axis of x and the axis of y itself, respectively.

Hamilton’s principle allows for the derivation of the fundamental equations of dynamics for honeycomb panels and equivalent panels of same form, by substituting the physical and geometric parameters, along with the constitutive relationships of each layer of the honeycomb panel, as well as the equivalent physical parameters, geometric parameters, and constitutive relationships of the equivalent panel into their respective dynamic fundamental equations, the following displacement-based fundamental equations are derived:

(5) $$\left\{\begin{array}{l} A_{1}^{k}\varphi_{x,xx}+(A_{2}^{k}+C^{k})\varphi_{y,xy}+C^{k}\varphi_{x,yy}-\\ \;a^{k}(\varphi_{x}+w_{,x})=I_{1}^{k}\overset{\cdot \cdot }{\varphi_{x}}\\ C_{1}^{k}\varphi_{y,xx}+(B_{1}^{k}+C^{k})\varphi_{x,xy}+B_{2}^{k}\varphi_{y,yy}-\\ \;b^{k}(\varphi_{y}+w_{,y})=I_{1}^{k}\overset{\cdot \cdot }{\varphi_{y}}\\ a^{k}(\varphi_{x,x}+w_{,xx})+b^{k}(\varphi_{y,y}+w_{,yy})+q=I_{0}^{k}\overset{\cdot \cdot }{w} \end{array}\right.$$

where I represents the generalized inertia; A, B, C, a, b denotes the coefficient, determined by integration.

The formula mentioned above indicates that if the coefficients of Equations (4) and (5) are rendered comparable, then for same boundary or initial conditions, the equivalent plate will provide the same solution as the original honeycomb plate. The elastic constant of the comparable plate can be determined by stiffness equivalence; the density of the equivalent plate can be ascertained by inertia equivalency. According to the above concepts, the physical parameters of the equivalent plate are as follows:

(6) $$\left\{\begin{array}{l} {\bar{E}}_{x}\\ {\bar{E}}_{y}\\ {\bar{G}}_{xz}\\ {\bar{G}}_{yz}\\ {\bar{G}}_{xy}\\ {\bar{\nu }}_{xy} \end{array}\right\}=\left\{\begin{array}{l} (e_{11}e_{22}-e_{12}^{2})/e_{22}\\ (e_{11}e_{22}-e_{12}^{2})/e_{11}\\ e_{44}\\ e_{55}\\ e_{66}\\ e_{12}/e_{22} \end{array}\right\}$$

(7) $$\left\{\begin{array}{l} e_{11}\\ e_{22}\\ e_{33}\\ e_{44}\\ e_{55}\\ e_{66} \end{array}\right\}=\left\{\begin{array}{l} \left\{\left[{\left(h+d\right)}^{3}-h^{3}\right]e_{f11}+h^{3}e_{c11}\right\}/{\left(h+d\right)}^{3}\\ \left\{\left[{\left(h+d\right)}^{3}-h^{3}\right]e_{f22}+h^{3}e_{c22}\right\}/{\left(h+d\right)}^{3}\\ \left\{\left[{\left(h+d\right)}^{3}-h^{3}\right]e_{f12}+h^{3}e_{c12}\right\}/{\left(h+d\right)}^{3}\\ \left(de_{f44}+he_{c44})/(h+d\right)\\ \left(de_{f55}+he_{c55})/(h+d\right)\\ \left\{\left[{\left(h+d\right)}^{3}-h^{3}\right]e_{f66}+h^{3}e_{c66}\right\}/{\left(h+d\right)}^{3} \end{array}\right\}$$

(8) $$\rho =\frac{d\rho_{f}+h\rho_{c}}{h+d}$$

(9) $$\begin{array}{ll} e_{c11}=e_{c22}=\frac{1}{1-\nu_{xy}^{2}}E_{x}, & \;e_{c12}=\frac{\nu_{xy}}{1-\nu_{xy}^{2}}E_{x}\\ e_{c44}=e_{c55}=G_{xz}=G_{yz}, & \;e_{c66}=G_{xy}\\ e_{f11}=e_{f22}=\frac{1}{1-\nu^{2}}E, & \;e_{f12}=\frac{\nu }{1-\nu^{2}}E\\ e_{f44}=e_{f55}=KG, & e_{f66}=G \end{array}$$

where $e_{fij}$ and $e_{cij}$ represent the stiffness coefficients of the surface material and the honeycomb sandwich in the previously mentioned coordinate system, respectively; $\rho_{f}$ and $\rho_{c}$ denote the mass densities of the surface material and the honeycomb sandwich, respectively; K is the influence coefficient, which can be taken as a value from 0 to 1 according to engineering practice or experiment, to determine the degree of influence of lateral shear on the surface skin layer, K = 1 when considering influence of lateral shear on the surface skin layer [23].

### 2.2. Equivalent Acoustic Excitation

This article performs similar computations on the sound pressure spectrum of a certain satellite, transforming the sound pressure spectrum into pulsating pressure power spectral density. The specific sound pressure spectrum is shown in Table 1.

This article examines noise excitation as a random pressure applied to the surface of a flat plate; it is consistently dispersed and fluctuates with time. Consequently, the sound pressure spectrum presented in the above table was transformed into pulsing pressure spectral density, with the specific conversion formula detailed as follows [5]:

(10) $$\varphi (f_{c})=\frac{P_{0}^{2}}{\Delta f_{c}}10^{0.1L_{P}}$$

where $f_{c}$ is the Center frequency of octave bandwidth, $P_{0}=2.0\times 10^{-5}\;\mathrm{Pa}$, $\Delta f_{c}$ is the bandwidth of the octave band, $L_{P}$ is the sound pressure level of octave band.

The challenge in predicting the vibration response of spacecraft structures to noise loads, from a mechanical standpoint, resides in the selection of damping parameter models and the consideration of fluid–structure coupling effects, the predictive relevance of reactions in frequency bands over 2000 Hz is typically negligible, this article computes the frequency range from 20 Hz to 2000 Hz. In this study, 1/3-octave sound pressure spectrum (Table 1) was converted into octave band data to serve as the input load for the structural finite element analysis. This simplification is justified by the modal characteristics of the structure under investigation, aiming to ensure the accuracy of global response predictions while effectively enhancing computational efficiency. Using the previous formula, the sound pressure spectrum under the noise test conditions within 2000 Hz presented in Table 1 was transformed into pulsating pressure spectral density, with the specific values indicated in Table 2.

### 2.3. Analysis of Peak Stress Caused by Random Excitation

Segalman [34] introduced a computational method for random vibration von Mises stress. Al-Bahkali [35] introduced failure and fatigue life due to random vibration in aircraft applications. The stress response to vibration subject to random excitation, derived from finite element analysis, is usually investigated by employing von Mises stress. The failure probability of isotropic structures during a particular period of time can be expressed as the probability of exceeding the von Mises stress limit. Finite element analysis can determine the RMS value $\sigma_{m}$ of von Mises stress.

The peak value of von Mises stress can be derived according to the 3σ principle.

(11) $$\sigma_{S}=3\sigma_{m}$$

### 2.4. Stress Analysis of Strain Rosette

The stress condition at the specified place is determined by attaching strain gauges during the noise testing procedure. Assuming that the surface strain measured at a specific place is $\varepsilon_{X}$, $\varepsilon_{Y}$, $\gamma_{XY}$, it is simple to derive the strain along any angle direction at an angle ϕ to $\varepsilon_{X}$ by strain analysis [36]:

(12) $$\varepsilon_{\phi }=\frac{\varepsilon_{X}+\varepsilon_{Y}}{2}+\frac{\varepsilon_{X}-\varepsilon_{Y}}{2}\cos2\phi +\frac{\gamma_{XY}}{2}\sin2\phi$$

The principal strain can be articulated as:

(13) $$\begin{array}{c} \varepsilon_{1}\\ \varepsilon_{2} \end{array}=\frac{\varepsilon_{X}+\varepsilon_{Y}}{2}\pm \sqrt{{\left(\frac{\varepsilon_{X}-\varepsilon_{Y}}{2}\right)}^{2}+{\left(\frac{\gamma_{XY}}{2}\right)}^{2}}$$

The principal strain direction is:

(14) $$tg2\phi_{0}=\frac{\gamma_{XY}}{\varepsilon_{X}-\varepsilon_{Y}}$$

The principal stress and maximum shear stress can be derived using the generalized Hooke’s law.

**Figure 2:**
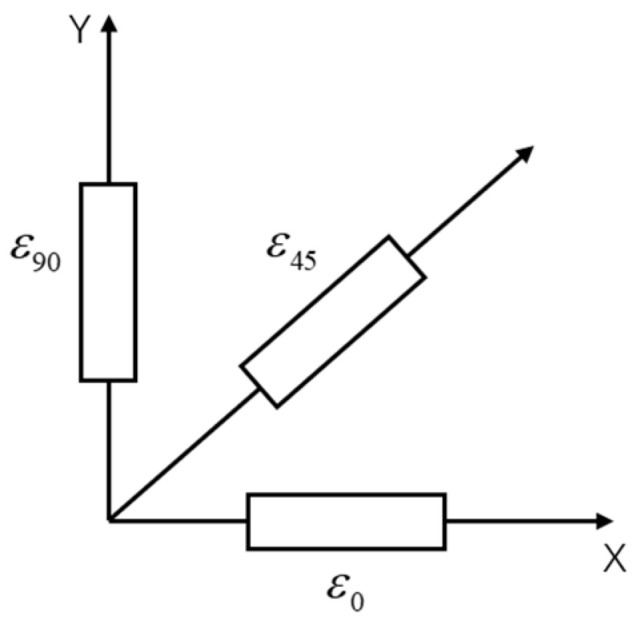
45°-3 right-angled strain rosette.

This article employs a 45°-3 right-angled strain rosette, with its structural configuration illustrated in Figure 2.

The formula for calculating strain is as follows:

(15) $$\begin{array}{c} \varepsilon_{1}\\ \varepsilon_{2} \end{array}=\frac{\varepsilon_{0}+\varepsilon_{90}}{2}\pm \frac{1}{\sqrt{2}}\sqrt{{\left(\varepsilon_{0}-\varepsilon_{45}\right)}^{2}+{\left(\varepsilon_{45}-\varepsilon_{90}\right)}^{2}}$$

The formula for calculating stress is as follows:

(16) $$\begin{array}{c} \sigma_{1}\\ \sigma_{2} \end{array}=\frac{E}{1-\nu^{2}}\left[\frac{1+\nu }{2}\left(\varepsilon_{0}+\varepsilon_{90}\right)\pm \frac{1-\nu }{\sqrt{2}}\sqrt{{\left(\varepsilon_{0}-\varepsilon_{45}\right)}^{2}+{\left(\varepsilon_{45}-\varepsilon_{90}\right)}^{2}}\right]$$

where E represents elastic modulus; ν represents Poisson’s ratio; $\varepsilon_{0}$, $\varepsilon_{45}$, $\varepsilon_{90}$ signify the corresponding strain magnitudes for 45°-3 strain rosette.

During strain testing, its weak signal renders it highly susceptible to external interference, significantly affecting the test results. Consequently, the strain value should be suitably calibrated in accordance with the experimental conditions during the testing procedure to approximate the genuine value [37].

## 3. Example of Simulation Analysis

### 3.1. Overview of Example

**Figure 3:**
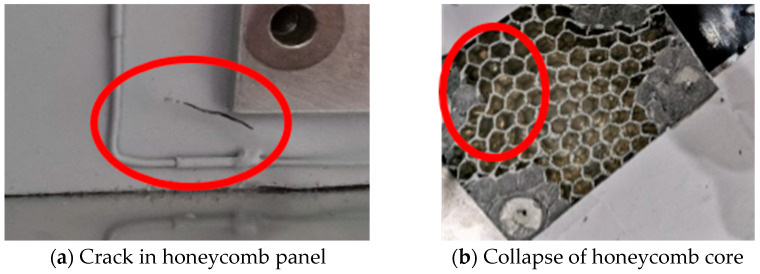
Experimental Phenomenon Diagram.

After conducting noise tests on a large satellite, it was found that a 1 cm long crack appeared on the surface of the honeycomb panel near the installation area of the solar wing bracket, and the internal honeycomb collapsed, as shown in Figure 3. The main reason for this problem appears to be the inadequate strength of the honeycomb core. During noise testing, the internal honeycomb core sustained damage, leading to honeycomb collapse and surface fissures. To validate this hypothesis, it is essential to do a response and stress analysis of noise excitation.

### 3.2. Establishment of Finite Element Model

**Figure 4:**
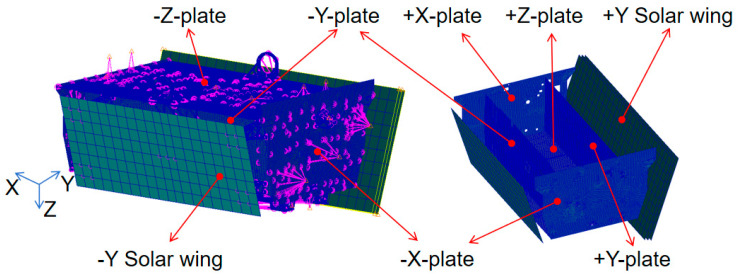
Finite element model of satellite.

The satellite employs a honeycomb panel structure, a 3D model was created employing 3D modeling software, subsequently followed by the partition into a finite element mesh, the honeycomb sandwich panel employs a previously mentioned equivalent theory for model equivalence. Simulations for small components are performed employing lumped mass or unstructured mass, with the corresponding finite element model illustrated in Figure 4.

The acoustic load analysis in this study is based on the diffuse acoustic field assumption, where pressure spectral densities are applied directly to the external wetted surfaces of the structure without explicitly solving the surrounding fluid domain. As an engineering simplification, this method does not account in detail for acoustic shielding effects due to geometry, instead assuming uniform pressure on all external surfaces. This approach is widely adopted for the preliminary vibration assessment of spacecraft structures, yielding conservative results with significant computational savings. Future work could involve employing boundary element methods or fully coupled acoustic-structural analysis to more accurately quantify the impact of shielding effects.

In the dynamic equation, the mass and stiffness matrix can be derived through finite element partitioning.

**Figure 5:**
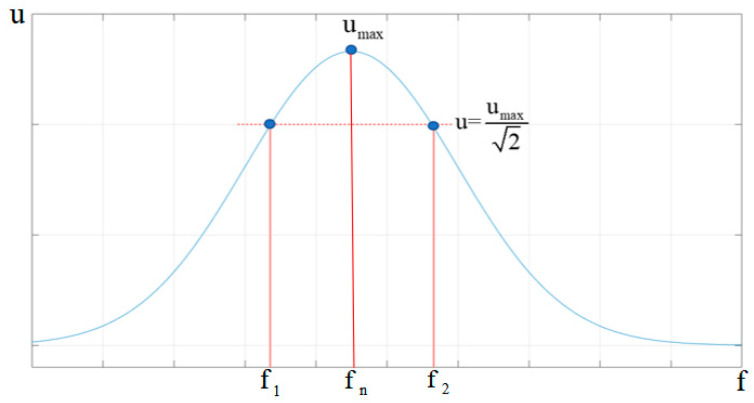
Measurement of the damping ratio by the half-power bandwidth method.

The damping ratio is a parameter of particular importance. Damping can only be determined according to experimentation. Using experimental modal testing techniques or specialized dynamic testing equipment, external force excitation was applied to the structure, and its amplitude–frequency response was measured. Processing of the measured data yields a frequency response curve (amplitude versus frequency), as presented in Figure 5.

The peak frequency f_n_ of the dynamic response is identified from the curve, the formula for calculating its damping ratio is as follows:

(17) $$\xi =\frac{f_{2}-f_{1}}{2f_{n}}$$

**Figure 6:**
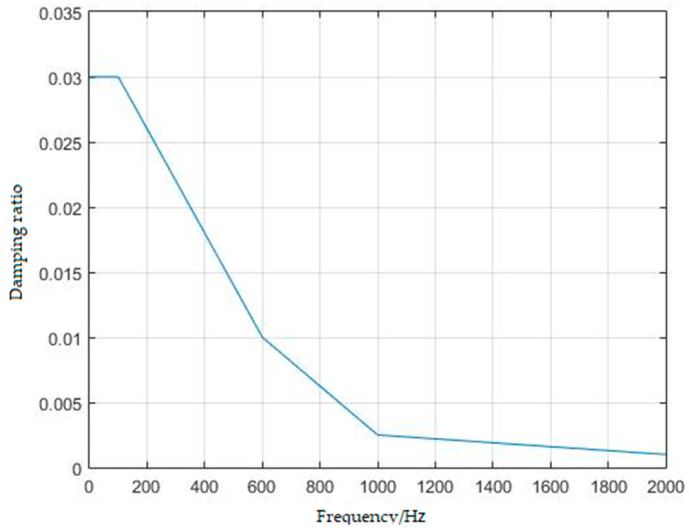
Damping parameter model.

Usually, the damping ratio for frequency response analysis is generally taken as a constant value of 0.03. The specific circumstances under which a damping ratio of 0.03 is appropriate are outlined below: low frequency random vibration environment. Flexible structures such as solar panels and large antennas exhibit low-frequency responses, typically between 20 and 200 Hz, in a dynamic analysis, a damping ratio of 0.03 is commonly employed under these conditions. The frequency range for random vibration analysis is generally 20–2000 Hz. In actual spacecraft vibration, as the frequency increases, damping decreases. Therefore, applying a universally fixed damping ratio of 0.03 does not yield accurate results. This article discusses the prediction of random vibration responses for different spacecrafts and the results of modal testing to develop a damping parameter model, as illustrated in Figure 6. Two different damping models were employed to compute the vibration response values in the results, and a comparative study as shown in Section 3.3 was performed to ascertain that the damping model chosen in this paper is more precise.

The previously mentioned finite element model was employed to analyze and predict the vibrational results subjected to acoustic excitation to validate the feasibility and precision of the simulation method.

### 3.3. Analysis of Vibration Response Results Subjected to Acoustic Excitation

**Figure 7:**
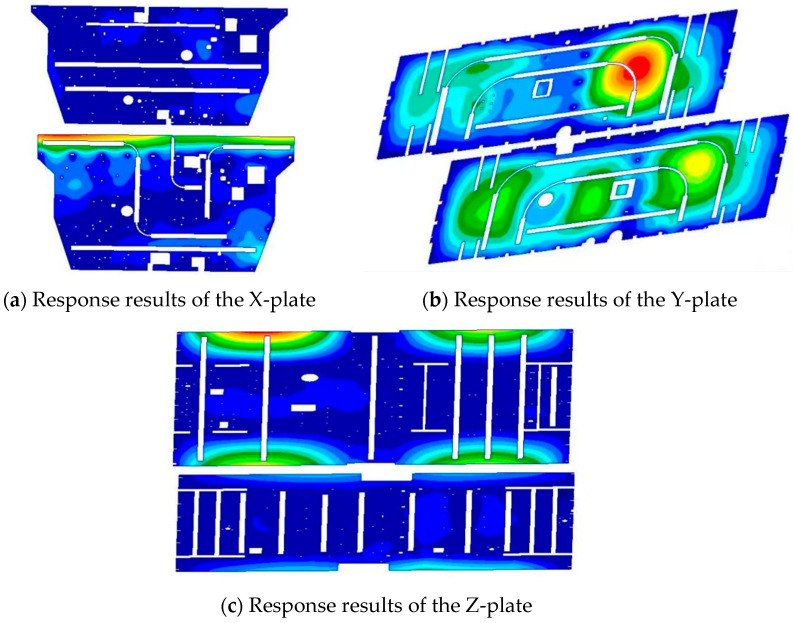
Simulation results of vibrational responses of several honeycomb panels subjected to noise excitation.

The previously mentioned approach for finite element simulation analysis provides the response of each honeycomb panel, as shown in Figure 7.

To provide a more accurate comparison with the experimental results, simulation data from nodes positioned identically to the vibration measurement locations in the experiment were extracted. The RMS of experimental results and simulation results of different damping parameters are presented in Table 3.

When compared to the test data, the damping model chosen in this article exhibits simulation values that correspond closely with the real test data, showing a maximum inaccuracy of 0.61 g when compared to the simulation data. The simulation result with a damping ratio of 0.03 shows a maximum error of 1.09 g. The precision of the simulation results and the viability of the simulation method have been confirmed by the data mentioned above.

### 3.4. Peak Shear Stress of Each Honeycomb Core Subjected to Acoustic Excitation

Comparison of the above data confirms that the method described in this research can precisely predict satellite vibrations subjected to noise excitation.

**Figure 8:**
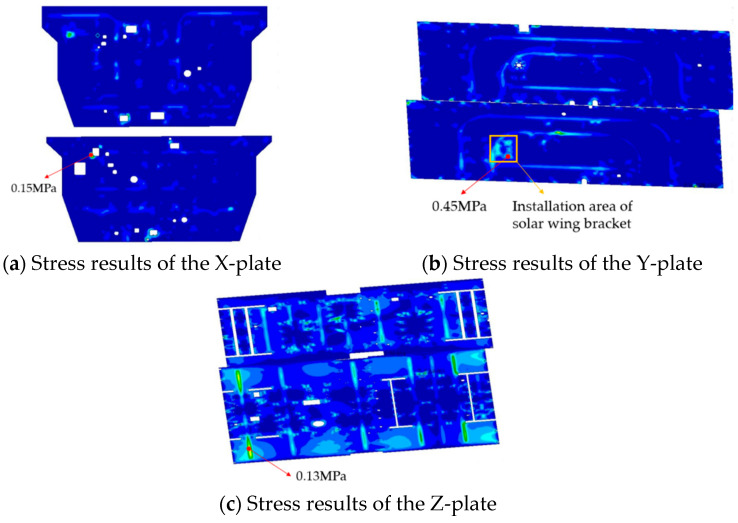
Results of stress simulations for each honeycomb panels.

To ensure the safe operation of satellites in orbit, this article conducted simulation analysis on the stress of each honeycomb panel, according to the 3σ principle, the peak shear stress of the honeycomb core is obtained as shown in Figure 8.

A summary of the maximum stress for each honeycomb panel is provided in Table 4. The peak stress of the Y plate is 0.45 MPa, positioned at the identical location as the honeycomb collapse observed in the experiment. The stress limit of sparse honeycomb is 0.44 MPa, the peak stress of the Y plate exceeded the stress limit of comb honeycomb, causing damage to the honeycomb core.

To ensure the safety of the satellite structure and meet the requirements of satellite weight reduction, the comb honeycomb will be replaced with local dense honeycomb, and stress and response simulation analysis will be conducted.

### 3.5. Stress and Response Simulation Results of Local Dense Honeycomb Scheme

Based on the finite element simulation results, the honeycomb core in the region of elevated stress was modified to a denser configuration. The specific parameters of sparse honeycomb structure and dense honeycomb structure are shown in Table 5.

**Figure 9:**
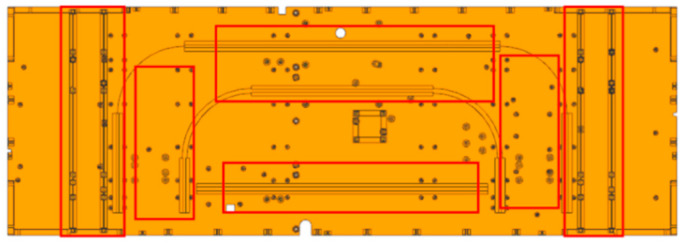
Schematic depiction of altering the position of dense honeycomb.

Considering the −Y board as a case study, the stress calculation results indicate that the area with elevated stress, highlighted in the red box in Figure 9, is modified to a dense honeycomb structure, and employ the above simulation method to recalculate.

**Figure 10:**
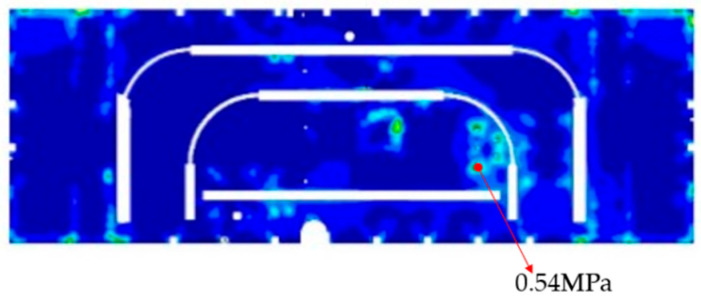
Stress profile of the −Y plate.

The above finite element simulation approach was used for stress analysis to obtain the stress profile of the −Y plate as illustrated in Figure 10. The peak stress measurement is 0.54 MPa, located at the sail support connection, consistent with the prior position. All components of the honeycomb core can satisfy design specifications, remaining much less than the stress limit of 2.34 MPa for dense honeycomb.

The maximum stress of the other honeycomb panels is presented in Table 6 and the honeycomb cores of each panel satisfy their design specifications.

For the −Y board, the location of the maximum stress remains consistent across different honeycomb structures. The peak stress measurement of the dense honeycomb structure is 0.54 MPa, and the peak stress measurement of sparse honeycomb structure is 0.44 MPa, the adoption of the dense honeycomb structure results in a slight increase in the maximum stress compared to the sparse honeycomb structure.

**Figure 11:**
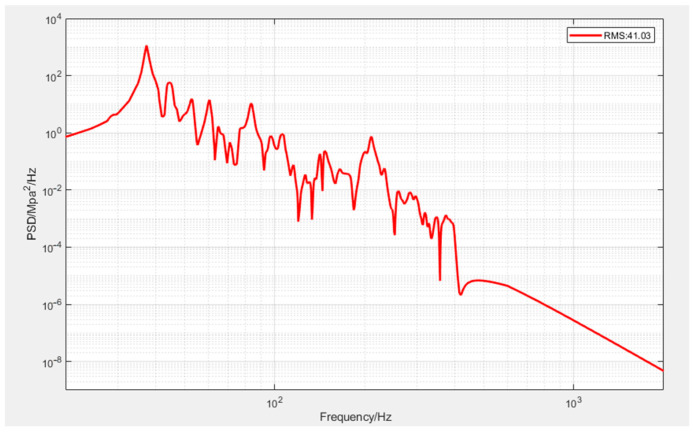
PSD map of skin stress at the location of honeycomb collapse.

**Figure 12:**
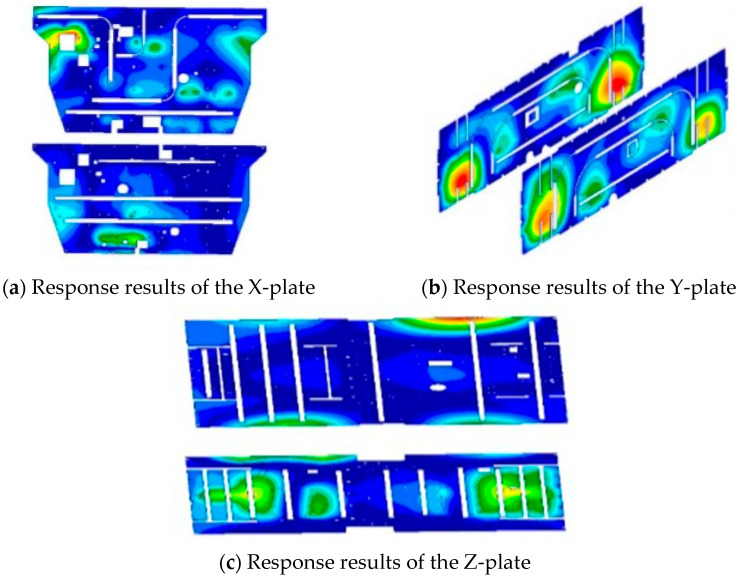
Results of response simulations for each honeycomb panel following the alteration of the plan.

Due to the difficulty to properly quantify the shear stress of the honeycomb core during the acoustic testing procedure, consequently, by attaching a strain rosette to the skin’s surface corresponding to the honeycomb’s collapse, the stress conditions at that specific location may be evaluated. To validate the precision of the simulation method presented in this article, the stress curve of the skin at the site of honeycomb collapse derived from the simulation is illustrated in Figure 11 below:

The previously mentioned finite element method was employed for noise response analysis, with the response of each honeycomb panel shown in Figure 12.

## 4. Noise Testing

### 4.1. Overview of Noise Testing

**Figure 13:**
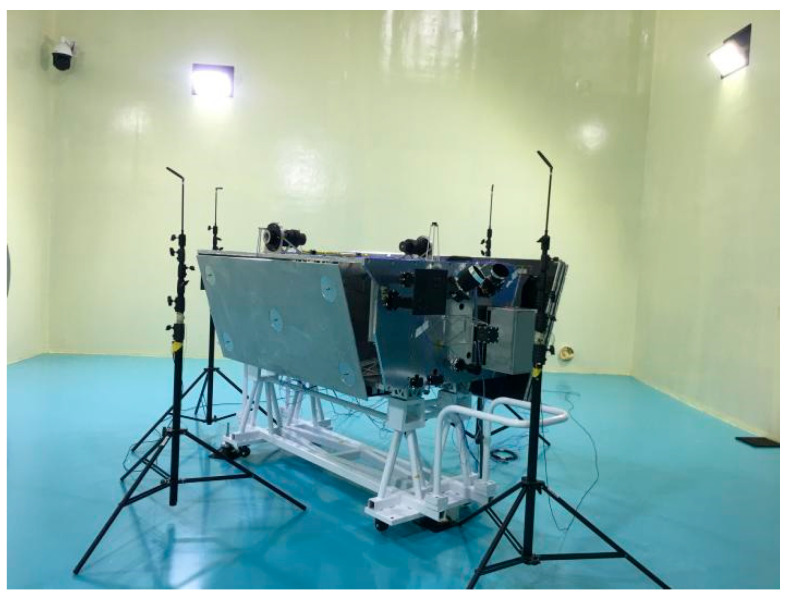
Picture of noise test site.

Following the modification of the honeycomb panel, a subsequent noise test was performed, with the test site shown in Figure 13 below.

The satellite was positioned in the reverberation chamber, with acceleration sensors attached at designated locations to monitor the vibration response subjected to acoustic excitation. The shear stress of the honeycomb core cannot be directly measured; therefore, stress monitoring is conducted on the skin at the damaged location of the honeycomb panel. Strain gauges are attached to the skin of the honeycomb panel at the location of the original panel’s collapse, and the strain at that position is monitored during the noise testing process to assess its stress condition.

### 4.2. Stress Results of the Noise Test

**Figure 14:**
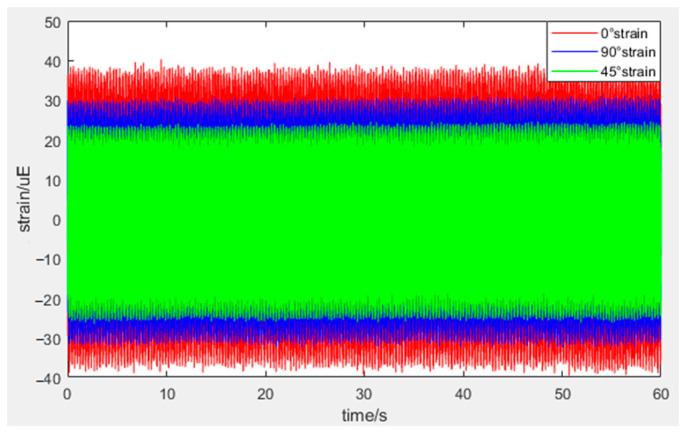
The time-domain signal of the strain.

During the noise testing, stress gauges were attached to the honeycomb panel surface at the locations correlating to the honeycomb collapse. The time-domain signal of the strain measured during the test is shown in Figure 14:

According to the strain test results, the stress values were computed using Formulas (15) and (16), with a comparison of the test and simulation results presented in Table 7.

### 4.3. Response Results of the Noise Test

The RMS of test response results and simulation results at different locations are presented in Table 8.

**Figure 15:**
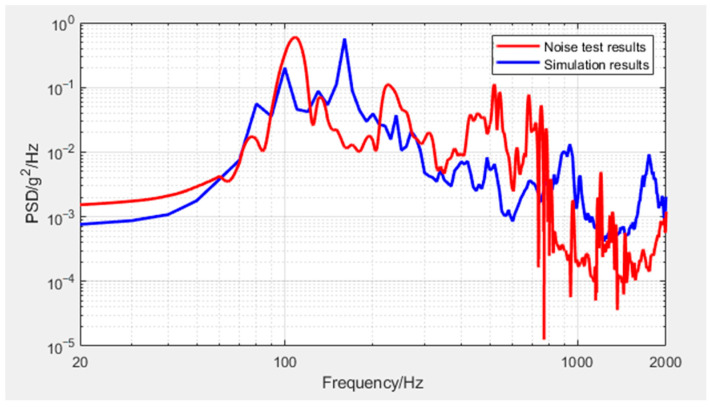
Curves of the noise test results as well as the simulation results of magnetic torque.

Taking the magnetic torque as an example study, Figure 15 presents the noise test results as well as the simulation results curves.

The accuracy of the simulation method and the rationale for locally increasing the density of honeycomb panels were further validated by comparing and analyzing the simulation results of noise response with experimental results. However, a discrepancy persists between the experimental and simulated results. The main sources of error are identified as follows:

1. Model simplification errors: This study employs an equivalent honeycomb panel model, which inherently approximates the actual structure and thus introduces minor discrepancies.
2. Regarding the acoustic load application method (without fluid model): This study employs the “diffuse acoustic field” assumption, the complex fluid–structure interaction problem, which involves solving the coupled acoustic wave equation and structural mechanics, is simplified to applying a uniform pressure spectrum to the wetted surfaces (those exposed to the acoustic field). This approach significantly reduces computational cost while maintaining engineering accuracy.
3. Uncertainty in material parameters: The impact of testing errors or batch variations in the material properties (equivalent model parameters, damping parameter, etc.) used in the simulation is analyzed.
4. Deviation in boundary conditions and loading: The differences between the ideal boundary/loading conditions in the simulation and the actual conditions that are difficult to perfectly replicate in experiments are compared.
5. Measurement errors: Errors including sensor noise, the accuracy of the data acquisition system, and identification errors in image processing are included. The sensor used in this experiment is the acceleration sensor model 8763B500 from Kistler Company.

In this study, while the overall global response (RMS values) shows good agreement, the frequency-domain agreement, particularly for peak locations and higher frequencies, is less perfect. The noticeable differences in the frequency of the PSD maxima are primarily attributed to imperfections in the boundary conditions of our Finite Element Model. The degradation in correlation at higher frequencies can be explained by two main factors: model fidelity limitations and damping model uncertainty. The good agreement in RMS values (Table 7) demonstrates that our methodology is effective and reliable for predicting global vibration load levels.

Post-experiment, the honeycomb panel’s surface was examined, revealing no indications of damage, and the findings of the characteristic level tests conducted before and after the noise test were consistent, signifying that the satellite body remained undamaged.

## 5. Conclusions

This paper develops a finite element analysis model for the vibration of satellite structures subjected to acoustic excitation. Equivalent calculations were conducted on the honeycomb sandwich panel construction to determine its response and stress, transform acoustic excitation into random pressure exerted on the satellite’s flat surface, and studied the impact of damping parameters on simulation results. In reaction to the phenomenon of damage to the honeycomb panel structure during noise testing of a satellite, the vibration response results under noise excitation were analyzed comparatively. The damping model employed in this research demonstrated superior predictive accuracy for response results compared to the traditional constant damping model, with a maximum error of 0.61 g. Upon checking the stress of the honeycomb sandwich panel, it was determined that the maximum stress at the site of the honeycomb core failure was 0.45 MPa, exceeding the stress limit of the sparse honeycomb core, hence identifying the reason of the honeycomb panel damage. According to simulation results, the honeycomb panel was locally modified to a dense honeycomb structure, and its maximum stress subsequently satisfied the design specifications. Noise testing was conducted once more, ensuring that the difference between the stress result and the simulation results of the skin at the site of honeycomb collapse is less than 10%, and the maximum difference between the noise test response results and the simulation results is 0.58 g, verifying the feasibility and accuracy of the satellite stress and response analysis simulation method subjected to acoustic excitation in this article, the simulation indicates that the maximum shear stress of the honeycomb core is 0.54 MPa, far less than the stress limit of the dense honeycomb, which solves the issue of honeycomb collapse. Simultaneously, employing a dense honeycomb structure locally can guarantee the satellite’s safe operation in orbit.

The paper establishes a finite element simulation model using an equivalent honeycomb model and empirical damping model, transforming the sound pressure spectrum into pressure power spectral density, converting noise excitation into random excitation, and implementing it on the surface of the satellite finite element model. A variable damping model instead of fixed value damping is adopted; the response and stress results subjected to noise excitation are analyzed and tested.

This analytical method serves as a reference for subsequent research. The simulation method proposed in this work enables a dynamic analysis of a large spacecraft and space stations during rocket launch phases. Our forthcoming work will prioritize the following aspects: composite material applications, variable damping research, and nonlinear dynamic response analysis, the integration of machine learning for faster parameter optimization or coupling with high-fidelity commercial solvers.

## Figures and Tables

**Table 1:** Conditions for satellite noise testing.

Center Frequency of Octave Bandwidth (Hz)	Sound Pressure Level (dB)	Test Tolerance (dB)
31.5	119.5	−2/+4
63	132.5	−1/+3
125	136	
250	136.5	
500	135	
1000	128.5	
2000	123.5	
4000	119.5	−6/+4
8000	115.5	
Total sound pressure	141.5	−1/+3
Test duration	1 min	

**Table 2:** Power spectral density.

Frequency (Hz)	Power Spectral Density (Pa^2^/Hz)
20~63	16.00
63~125	159.67
125~250	181.62
250~500	101.07
500~1000	35.78
1000~2000	4.00
Test duration	1 min

**Table 3:** Comparison data table of noise test results and simulation results.

Number	Sensor Location	Linked with Honeycomb Panel	Value of Noise Test (/g)	Value of Noise Simulation (Damping Model) (/g)	Value of Noise Test (0.03) (/g)
1	Magnetic torque	+Z	5.48	5.03	4.56
2	Antenna 1	+X	3.93	3.38	3.05
3	Gyroscope	+Y	7.21	6.60	6.12
4	Amplifier	−X	3.57	3.04	2.72
5	Flywheel	−Y	4.68	4.35	3.86
6	Antenna 2	−Z	4.67	4.10	4.02

**Table 4:** Maximum stress of each honeycomb panel (Sparse honeycomb).

Number	Honeycomb Panel	Value of Maximum Stress Simulation/MPa
1	±X	0.15
2	±Y	0.45
3	±Z	0.13

**Table 5:** Parameters of different honeycomb structure.

Number	Honeycomb Structure	Dimensional Parameter	Ultimate Stress/MPa
1	Sparse honeycomb	5 × 0.03	0.44
2	Dense honeycomb	3 × 0.05	2.43

**Table 6:** Maximum stress of each honeycomb panel (Dense honeycomb).

Number	Honeycomb Panel	Value of Maximum Stress Simulation/MPa
1	±X	0.25
2	±Y	0.54
3	±Z	0.21

**Table 7:** Comparison of the test and simulation stress results.

Number	Location of Strain Rosette	Test Value/MPa	Simulation Value/MPa	Error/%
1	Surface at the site of honeycomb collapse	45.05	41.03	8.92

**Table 8:** Comparison data table of noise test results and simulation response results.

Number	Sensor Location	Linked with Honeycomb Panel	Value of Noise Test (/g)	Value of Noise Simulation (Damping Model) (/g)	Errors Between Simulation and Empirical Measurement (/g)
1	Magnetic torque	+Z	5.70	5.12	0.58
2	Antenna 1	+X	4.26	4.02	0.24
3	Gyroscope	+Y	7.89	7.33	0.56
4	Amplifier	−X	3.32	2.93	0.39
5	Flywheel	−Y	5.03	4.63	0.40
6	Antenna 2	−Z	5.12	4.65	0.47

## Data Availability

The original contributions presented in this study are included in the article. Further inquiries can be directed to the corresponding author.
